# Characteristics and motivations of absconders from forensic mental health services: a case-control study

**DOI:** 10.1186/1471-244X-14-91

**Published:** 2014-03-27

**Authors:** Treena Wilkie, Stephanie R Penney, Stephanie Fernane, Alexander I F Simpson

**Affiliations:** 1 Centre for Addiction and Mental Health, 1001 Queen Street West, Toronto, ON, Canada M6J 1H4; 2 University of Toronto, Toronto, ON, Canada

**Keywords:** Absconding, Risk, Forensic mental health

## Abstract

**Background:**

Absconding from hospital is a significant health and security issue within psychiatric facilities that can have considerable adverse effects on patients, their family members and care providers, as well as the wider community. Several studies have documented correlates associated with absconding events among general psychiatric samples; however, few studies have examined this phenomenon within samples of forensic patients where the perception of threat to public safety in the event of an unauthorized absence from hospital is often higher.

**Methods:**

We investigate the frequency, timing, and determinants of absconding events among a sample of forensic psychiatric patients over a 24-month period, and compare patients who abscond to a control group matched along several sociodemographic and clinical dimensions. We explore, in a qualitative manner, patients’ motives for absconding.

**Results:**

Fifty-seven patients were responsible for 102 incidents of absconding during the two year study window. Forensic patients who absconded from hospital were more likely to have a history of absconding attempts, a diagnosed substance use disorder, as well as score higher on a structured professional violence risk assessment measure. Only one of the absconding events identified included an incident of minor violence, and very few included the commission of other illegal behaviors (with the exception of substance use). The most common reported motive for absconding was a sense of boredom or frustration.

**Conclusions:**

Using an inclusive definition of absconding, we found that absconding events were generally of brief duration, and that no member of the public was harmed by patients who absconded. Findings surrounding the motivations of absconders suggest that improvements in therapeutic communication between patients and clinical teams could help to reduce the occurrence of absconding events.

## Background

Absconding from psychiatric facilities is a significant health and security concern especially for forensic patients who are legally mandated to remain in a secure setting. Incidents of absconding can have considerable adverse effects on the community, hospital and patients alike. There is a large social and economic cost of absconding; for example, police are found to be involved in returning between 13% and 33% of absconders to hospital [1]. Absconding can also have detrimental effects on the hospital and care providers, while relatives of patients and community members may experience a decreased sense of confidence in the psychiatric services being provided [1,2]. When considering forensic populations in particular there is often a heightened perception of risk to public safety, highlighted by recent calls for greater restrictions on those with psychiatric illnesses who are in conflict with the law (e.g., in Canada, Bill C-54 [Not Criminally Responsible Reform Act]). There are currently no empirical findings directly comparing the rate of interpersonal violence or offending among civil versus forensic psychiatric patients who have absconded from hospital.

The impact of absconding may be most acutely felt by patients themselves as these incidents may slow recovery by prolonging hospitalization and interrupting treatment efforts. Also, the literature suggests an association between self-harm behaviors and absconding. A recent review found that 25% of all suicides among inpatient psychiatric clients over a 10-year period in England and Wales took place after patients had absconded from the ward [3], and older studies have reported comparable figures [4-6]. Dickens and Campbell [7] reported that 16% of absconding events in their sample of psychiatric inpatients in the U.K. involved serious adverse outcomes including self-harm and victimization. In contrast, the vast majority of studies report very low base rates of offending behavior and violence towards others occurring during a patient’s absconsion (e.g., for violence: 1.6% [8]; 1.4%, [9]), including absconders who are forensic patients (2.8% [10]; 3.2% [11]; 4.6% [12]; 4.4% [13], for all absconding events involving interpersonal violence). Within samples of individuals deemed not criminally responsible for their offenses due to mental illness, there is evidence to suggest that those with a history of absconding are more likely to be re-arrested following their hospitalization [14,15].

The frequency of absconding is difficult to distill from the literature, given the varying definitions and measurement of this behavior across studies. That said, most studies appear to adopt a broad definition of the behavior, commonly defining absconding as leaving the hospital without permission [16-18] and including a failure to return from an authorized leave [7,8,12,19-21]. In their systematic review of absconding, Bowers and colleagues [1] reported the mean rate of absconding for general psychiatry (excluding forensic services) was 12.6% of all patients ‘at risk’ (defined as the total number of inpatients at the beginning of the study period plus the number of those admitted in the course of the study), with a range of 2-44%. Studies from the secure forensic hospitals in the U.K. report lower prevalence rates of between 1-4% of all admissions [10-12,22], and adopt similar definitions of absconding (i.e., any unauthorized absence from the secure hospital, including an escape from within the perimeter walls; 10,13,22). Given the negative impact of absconding events, further investigation is needed to understand the determinants and outcomes of absconding events, and the characteristics and motives of forensic patients who abscond. This could lead to more effective management of risk, by helping to refine models of decision-making surrounding leave and privilege authorizations, and ultimately, readiness for reintegration into the community.

## Characteristics and motivations of patients who abscond

### General psychiatric samples

Several studies have investigated the characteristics of patients who abscond from non-forensic psychiatric facilities, and have compared them to non-absconding patients on various demographic and clinical characteristics. Studies reveal that absconders tend to be young, male, and diagnosed with schizophrenia [1,7,19,23,24]. Andoh [20] found that absconders were more likely to have been referred to hospital by police, have employment problems, infrequent visits from family or friends, and a history of alcohol abuse. Having a history of absconding is found to significantly and substantially increase the likelihood of further absconding [16,19], while legal status (i.e., involuntary) within general psychiatric populations has also been found to increase patients’ risk of absconding [3,7,17,20,24].

Dickens and Campbell [7] compared a group of 88 absconders with a control group of non-absconders (*n* = 1378) over a three year period. They found no significant differences for sex, ethnicity or length of admission. However, absconders were significantly younger, more likely to be detained upon admission and more likely to be unmarried than a control group of non-absconders. Half of the absconding events lasted between two and sixteen hours, and police services were involved in returning just under one-quarter (23.6%) of absconders. Other studies have similarly documented that most absconding events are short in duration (e.g., < 24 hours; [12,24]), and occur while patients are already off the unit or hospital grounds with permission [7,21,25].

The motivations of absconding behavior have not been well documented, with only a small number of studies inquiring with clinical staff or patients themselves as to the reasons for the incident. Bowers et al. [8] conducted interviews with patients who absconded from 12 acute admission psychiatric wards in London. Patient narratives described boredom, feeling confined, frustration, and needing to complete a task (e.g., related to household responsibilities) as motivations for absconding. Although psychiatric symptoms contributed to the decision to abscond in some cases, these patients were also able to provide non-illness related motives for their behavior. These authors highlighted that over half of the patients who absconded had previously voiced their intentions to nursing staff. Muller [18] reported on patient-identified reasons for absconding, which included variables related to breakdowns in treatment (e.g., poor doctor/patient alliance, medication issues and active symptomatology), as well as family problems or a lack of family involvement. Falkowski and colleagues [26] found that 19% of absconders cited being disturbed by other patients, while 39% of absconders referred to disliking aspects of the hospital as a salient factor motivating their decision to abscond (e.g., disliking the staff, the ward itself, or the food). The literature on consumer experiences of acute psychiatric units frequently documents fear and safety concerns, both in terms of other patients’ perpetrating actual violence, but also feeling threatened, harassed, or having property stolen [27-29]. These concerns have been linked to patients’ decisions to abscond from inpatient units [30].

### Forensic psychiatric samples

The few studies investigating forensic samples show similar trends to the findings described in civil samples. Those who abscond from forensic settings are more likely to be young, male, and diagnosed with a psychotic disorder [2,11,13], and present with a history of absconding [10]. Brook et al. [10] demonstrated that, over the course of the 12 months preceding the absconding event, absconders were more likely to be involved in property damage, assaultive behavior, self-harm, and to be non-compliant with treatment. Absconding was also found to be associated with a worsening mental state and/or a recent exacerbation of symptoms [2,10]. Having an offence history of violence, bodily harm or wounding was found to be more common among those who abscond as compared to non-absconders [10,31].

In light of the perceived public safety concerns related to absconding from secure hospitals, of relevance within forensic samples is the duration and method of absconding, as well as the prevalence of violent or offending behaviors committed while the person is absent from hospital. Here too the findings appear similar to general psychiatric settings, in that the length of time a patient is reported to be missing is often under 24 hours [11,12,22], and the absconsion occurs after the patient has been granted permission to leave the ward or hospital grounds [10,12,13]. Incidents of absconding from inside locked units and secure hospitals are found to be extremely rare; for example, Moore [22] documented just 12 incidents of unauthorized absence occurring from within the perimeters of the three English Special Hospitals combined (i.e., Ashworth, Broadmoor, and Rampton) between 1989 and 1994. Similarly, acts of violence occurring during a patient’s absconsion are infrequent; as noted above, rates range from 3-5% of all absconding incidents.

One study has inquired into the motivational factors driving forensic patients’ absconding behavior. Dolan and Snowden [11] found that most patients who had absconded simply cited a desire to be at liberty, even for a short duration, as the main reason for their behavior. These authors noted that most of these patients perceived themselves to be in an unbearable position (i.e., facing an indeterminate length of time in hospital). Only one absconding incident out of 31 appeared driven by psychotic motivations.

## The current study

The current study expands upon the existing literature by describing the frequency, timing, characteristics and predictors of absconding behaviors among a sample of forensic patients within a large psychiatric hospital in Toronto, Canada. We identify the demographic, clinical and legal characteristics of patients with and without absconding incidents, and assess whether there are significant differences between these groups. We describe the characteristics of absconding events themselves, including the method by which they occur, their duration and repetition. We review relevant events transpiring during the absconding event, as well as in the month prior. Lastly, we examine variables shown to be associated with absconding in the existing literature, in addition to those relevant within forensic contexts (e.g., risk estimates for future violence), with the aim to identify those factors that are most strongly and uniquely predictive of this behavior. Information regarding patient motives for absconding was collected to describe different absconding profiles that may be reliably differentiated from each other.

## Methods

### Setting

This study was conducted at a large urban psychiatric hospital in Toronto, Canada. The forensic program within the hospital is the largest provider of forensic mental health services in the province of Ontario, comprising 180 inpatient beds divided between four medium and four minimum secure units and serving approximately 250 outpatients living in the community. The majority of patients have been found Not Criminally Responsible on account of Mental Disorder (NCRMD) and are under the auspices of the Ontario Review Board (ORB). The ORB is responsible for annual reviews of the status of every person under its jurisdiction and making ultimate decisions regarding the least restrictive placement of the individual (i.e., continued detention, conditional or absolute discharge from hospital) vis-à-vis public safety. In Canada, both summary convictions and indictable offenses are eligible for a NCRMD defense, resulting in a broad range of offenses ultimately receiving this designation. A current snapshot of our patient population (2012-2013) revealed that the most common index offense was assault (58%), followed by uttering threats (20%) and weapons-related charges (15%). Twelve percent of patients had been charged with a sexual offense, while 13% were charged with murder (7%) or attempted murder (6%). Eight percent had only a non-violent (e.g., property) offense for which they were found NCRMD.

At the time that this study was conducted, the process of granting privileges and leave from the hospital was based on clinical team discussions, and ultimately decided by the patient’s psychiatrist and approved by the unit manager. Clinical variables, including the patient’s current mental status and their community reintegration needs/readiness were considered when deciding whether and what level of privilege was granted. The upper limit of the privilege (i.e., amount of community access permitted) is set by the patient’s ORB disposition.

### Design

A case-control design was used. All current forensic patients who had at least one incident of absconding from hospital within the previous 24-months were identified (*n* = 57). An equally-sized control group was formed in order to conduct group-based comparisons along specified demographic and clinical variables. The control group was individually matched on age (within five years, and most [73%] within three years), sex, and security level within the hospital (residing on the same unit as their absconding counterpart, or on a unit with the same level of security). We also took care to ensure that no patients in the control sample had any history of absconding; otherwise, it is possible that our two groups would have simply differed on the timing of their absconding behavior rather than its presence or absence. With just one exception, every patient in the absconding group was matched successfully to a control patient in this manner.^a^ Consistent with prior research, we defined absconding as any unauthorized absence from the hospital. This included breaching the security of an inpatient unit, accessing hospital grounds or the community without permission, or being absent for longer than permitted. The study was approved by the institutional ethics review board prior to the commencement of data collection.

### Measures

A comprehensive coding scheme was developed to gather all relevant demographic and clinical information for each patient group. For patients who had an incident of absconding in the past 24 months, additional data pertaining to the month prior to the event (e.g., medication change or non-compliance, change in mental status, substance use, voiced ideation/intent to abscond), events transpiring during the unauthorized absence (e.g., involvement in or experience of violence, substance use), as well as characteristics of the absconsion itself (e.g., method of leave, duration, location traveled to, form of return to hospital) were recorded. All data were collected from the patient’s health record, including assessment and treatment reports, legal documents, as well as daily progress notes completed by nursing staff and other members of the clinical team. Data pertaining to absconding events in particular were collected and cross-referenced across three separate sources (daily progress notes, incident reports, and required email communications when a patient absconds). Information pertaining to patient motivations was also collected from the daily progress notes. These notes summarized the interaction that took place with the patient upon their return to the unit, including patients’ responses to being asked directly about why they absconded. Notes from biweekly meetings occurring between the patient and his or her attending psychiatrist were also accessed, as they often contained additional information about the absconding incident.

The Historical, Clinical and Risk Management-20 (HCR-20; [32]) was used to compare absconding versus non-absconding patients, as well as to predict the occurrence of an absconding incident. The HCR-20 is a 20-item violence risk assessment scheme for use with adults who have a history of violent behavior as well as mental illness and/or personality disorder. The items appearing on the HCR-20 may be grouped thematically into historical/static risk factors, clinical/current concerns, and future-oriented/risk management variables, and are coded on a 0 (not present), 1 (possibly or partially present), and 2 (definitely present) point scale.

The Psychopathy Checklist, Revised (PCL-R; [33]) was used for similar purposes. It is a 20-item symptom construct rating scale designed to measure the interpersonal, affective, and behavioral characteristics of psychopathic personality disorder in adults. The items appearing on the PCL-R are scored on a 0, 1, 2 scale reflecting trait presence and severity.

### Data analysis

Statistical tests of difference (*t*-test [Mann-Whitney *U* for variables with non-normal distributions], *χ*^2^) were used to compare the demographic and clinical profiles of absconding versus non-absconding patients, as well as between patients who absconded from secure/supervised settings versus non-directly supervised passes occurring on hospital grounds or in the community. We conducted Cox regression analyses to assess whether specified variables predicted the occurrence of an absconding incident, and if so, to identify the magnitude of association between each predictor variable and absconding. Predictor variables were tested in blocks of conceptually-related factors (e.g., clinical variables, risk-related variables). The Cox model is ideal as it allows for the inclusion individuals who have not yet experienced the outcome of interest (i.e., absconding) by the completion of the study. Lastly, qualitative thematic analysis of patient motives for absconding was undertaken to identify distinct profiles of absconding patients. To do this, the first three study authors (TW, SP, and SF) independently read all of the available clinical information surrounding a client’s absconsion (i.e., sources of information described in the ‘Measures’ section), including documentation of the patient’s self-reported motives. We then each rated what we judged to be the primary motivation(s) underlying the behavior; that is, the one or two variables that appeared to be functionally or causally related to their absconding. We subsequently met to discuss our ratings for each case. Based on this discussion, four distinct and non-overlapping profiles of absconding behavior were created, and each incident of absconding was assigned into one of the four groups. We were able to achieve perfect agreement at this stage; that is, for every case we agreed on the person’s group membership reflecting the primary motivation(s) driving the absconding behavior.

## Results

### Characteristics of absconding events

Fifty-seven patients were responsible for 102 incidents of absconding during the two year study window. This corresponds to a rate of 14.4%, calculated as the number of patients absconding (57), divided by the number of patients at risk (395; corresponding to the total number of inpatients at the beginning of the study plus the number admitted over the course of the study; as per [34]). Thirty-five patients had single incidents, while 22 patients absconded on two or more occasions. There were fewer incidents during the months of February (*n* = 2) and March (*n* = 5), as compared to the warmer months of June (*n* = 11) and July (*n* = 10), as well as during the holiday season in December (*n* = 13). Across the eight inpatient units studied, there were substantially more absconding events occurring off of the minimum secure units (92% of all events) as compared to the medium secure units (8%). This is to be expected, given that the level of hospital grounds and community access is significantly greater for patients residing on minimum secure units.

Descriptive data pertaining to clinically relevant events occurring in the month prior to an incident of absconding are presented in Table 1, while characteristics of the absconding events are presented in Table 2. There were changes in medication as well as the patient’s mental status/symptoms in the month prior to one-third of absconding incidents. Stressors/adverse events were documented in 39% of incidents; the source of the stress was most commonly related to the patient’s annual review board hearing. Eighty percent of incidents were characterized by privilege level changes (increases or decreases) in the month prior, while just over half were preceded by one or more documented instances of non-compliance with the privilege level. This most commonly involved the patient failing to sign in/out on time for their pass, or arriving back to the unit late (but before a formal absconding protocol was initiated, typically in the range of 5-10 minutes). In approximately one-third of incidents the patient was noted to express thoughts of absconding from the hospital, and in some cases directly stated their intention to abscond to nursing staff or other members of the treatment team.

**Table 1:** Events occurring in the month prior to absconsion

	**% of all leaves (*N*** **= 102)**
Medication change	39
Noncompliance with medication	26
Change in symptoms/mental status noted	32
Stressful/adverse event noted	39
Suicidal ideation expressed	4
Absconding ideation expressed	31
Attempted absconding	10
Noncompliance with privileges/passes	54
Change in privilege level	80
Engagement in violence	22
Engagement in substance use	14

**Table 2:** **Characteristics of absconding events (*N*** **= 102)**

	**Mdn**	**M**	**SD**	**Min**	**Max**
**Duration (hours)**	**10.50**	**50.67**	**126.60**	**0.33**	**912.00**
		**N**		**%**	
Method					
Off locked unit		14		13.7	
From staff accompanied outing		13		12.7	
From unaccompanied hospital pass		44		43.1	
From unaccompanied community pass		27		26.5	
Location during leave					
Within city limits, outdoors		42		41.2	
Own home		9		8.8	
Friends/family home		19		18.6	
Shelter		12		11.8	
Hospital grounds		6		5.8	
Another hospital		3		2.9	
Substance use (yes)		33		32.4	
Reoffense (yes)		1		1.0	
Violence – perpetrator		1		1.0	
Violence – victim		2		2.0	
Form of return					
Self		58		56.9	
Police		28		27.5	
Hospital staff		6		5.8	
Family member		7		6.9	

*Note*. Two outliers were removed in calculating the Duration variable: one incident that lasted 117 days, and another for 122 days.

The mean duration of absence was 2 days (*Mdn* = 10.5 hours), with a range of 20 minutes to 38 days (this excludes 2 patients who were absent for approximately 4 months each). Fifty-three percent of unauthorized absences were under 12 hours, while 67% were under 24 hours. The majority (70%) of absconding incidents occurred after the patient had already been granted permission to be on hospital grounds or its vicinity without direct staff supervision. In 22 incidents patients were still within close proximity to the hospital (i.e., on hospital grounds or within the downtown core where the hospital is located). In approximately one-quarter of events the patient was found to be either at their own homes (*n* = 9), or the home of a friend or family member (*n* = 19).

All patients who absconded returned to hospital. Police returned patients from 28 absconding incidents. In only one case had a patient engaged in some form of violence. This involved throwing a soft drink can at the hospital staff member who was trying to return him to hospital. One reoffense occurred in the form of a female patient who visited the home of the victim she had previously stalked on the basis of an erotomanic delusional belief. On two occasions, patients returned from their absconsion with visible injuries. One individual stated that he was mugged, while the other reported being beaten by police while being re-apprehended. Substance use was documented to have occurred in 32% of absconding incidents.

### Absconders versus controls

Means and standard deviations for the demographic, clinical and legal characteristics of the sample are presented in Table 3, as well as statistical tests of difference comparing absconders to matched controls. Patients who had one or more incidents of absconding spent significantly more time within the forensic mental health system (i.e., days under the auspices of the ORB) prior to their absconsion (*Mdn* = 1529), as compared the control group (*Mdn* = 1118; *U* = 2087.50, *p =* .005).^b^ For the absconding group, on average, 15 months elapsed between the time they were admitted for the index hospitalization and the time they first absconded (*M* = 15.07, *SD* = 20.01, *Mdn* = 8.00). There was a range of timing patterns depicting this behavior, however: 12% of patients were observed to abscond within 30 days of their admission, 46% within 6 months, and 15% waiting 2 years or more before absconding for the first time (range = 7 days to 8 years).

**Table 3:** Demographic, clinical and legal characteristics of absconders and controls

	**Absconders (*N*** **= 57)**		**Controls (*N*** **= 56)**	
	**M**	**SD**	**M**	**SD**
Age^*^	40.07	10.92	40.18	11.09
Days under ORB	2123.88_a_	1738.81	1317.70_b_	990.43
PCL-R total score	17.89	6.50	16.53	7.38
HCR-20 total score	25.53_a_	5.26	20.83_b_	5.62
	**N**	**%**	**N**	**%**
Sex (male)^*^	44	77.2	46	82.1
Ethnicity				
Caucasian	30	52.6	20	35.7
Afro-Caribbean	17	29.8	21	37.5
Asian	7	12.3	9	16.1
History of absconding*	32	56.1	0	0.0
Prior absconding attempts (yes)^†^	17	29.8	5	8.9
Diagnosis^†^				
Primary psychotic disorder	17	34.0	25	51.0
Comorbid psychosis + substance abuse	33	66.0	24	49.0
Personality disorder indicated (yes)	24	42.1	19	33.9
Index offense				
Violent	40	70.2	34	60.7
Non-violent	10	17.5	12	21.4
Sexual	7	12.3	10	17.9

*Note*. ORB = Ontario Review Board; PCL-R = Psychopathy Checklist, Revised; HCR-20 = Historical, Clinical, Risk Management-20.

*Denotes a variable that was matched across absconding and control groups (age, sex), or held to = 0 in the control group (history of absconding).

Means in the same row that do not share subscripts (a, b) differ at *p* < .01. ^†^For history of absconding attempts, χ^2^ = 9.89, *p* = .002; for diagnosis, χ^2^ = 2.94, *p* = .087.

Patients in the absconding group were more likely to have a history of unsuccessful absconding attempts (*χ*^2^ [1, *N* = 103] = 9.89, *p* = .002), as well as be diagnosed with a comorbid substance use disorder (most commonly alcohol or cannabis, *χ*^2^ (1, *N* = 99) = 2.94, *p* = .087). These patients were also estimated to be at higher risk for future violence, as indicated by scores from a structured professional risk assessment measure (HCR-20; [32]) completed by the individual’s psychiatrist and treatment team, *t* (105) = 4.47, *p* < .001.^c^ There were no significant differences between absconders and controls in terms of ethnicity, type of index offense, or personality dimensions (i.e., diagnosis of a personality disorder or level of psychopathic traits). When the index offense was further examined by the severity of violence involved, absconders were found to have fewer offenses involving serious violence as compared to non-absconders. Specifically, of those who had committed a violent offense, 55% of patients in the absconding group had committed an offense involving serious violence (i.e., resulting in a charge of murder, attempted murder, aggravated assault, or sexual assault), while 77% of the control group had committed one or more of these types of offenses, *χ*^2^ (1, *N* = 91) = 4.88, *p* = .027.

As noted, we adopted an inclusive definition of absconding behavior in order to capture all relevant events; however, this can result in a wide range and potentially heterogeneous pool of incidents being studied. In examining the group of absconding patients more closely, we found that all absconding incidents included in the study could be meaningfully captured by two broader categories, consistent with previous research in forensic settings [e.g., 22]: “opportunity makers” (those who absconded from a higher level of supervision [i.e., directly off a secure unit or from a staff accompanied outing; *n* = 16]) and “opportunity takers” (those who absconded while already on hospital grounds or in the community on an unsupervised pass [*n* = 38]). Notably, these two groups of patients did not differ on any of the variables listed in Table 3. Compared to patients with just a single incident of absconding, patients who absconded on multiple occasions had higher PCL-R (20.08 versus 15.59, *t* (39) = 2.33, *p* = .025) and HCR-20 (27.38 versus 24.38, *t* (53) = 2.12, *p* = .039) scores. They more frequently had histories of absconding attempts (*χ*^2^ [1, *N* = 49] = 4.41, *p* = .036), as well as comorbid substance use disorders (*χ*^2^ [1, *N* = 50] = 3.77, *p* = .052).

### Predictors of absconding

We next assessed the relation between specified clinical and risk-related variables (excluding those variables we had used for matching/control purposes, i.e., age, sex, and history of absconding) and absconding (coded present/absent) via Cox regression. As shown in Table 4, those with a comorbid diagnosis of a substance use disorder were more than twice as likely to abscond as compared to patients with a primary psychotic disorder in the absence of problematic substance use (OR = 2.38, *p* = .013). In contrast, those with a history of absconding attempts were not found to be significantly more likely to have an incident of absconding in the current study window, as compared to those without such attempts in their history. As shown in Table 5, higher scores on the HCR-20 were significantly associated with increased odds of absconding (OR = 1.11, *p* = .003). Indicators of personality pathology, either as noted by the treating psychiatrist or on a measure of psychopathic personality traits, were not related to the likelihood of absconding.

**Table 4:** Cox regression: clinical predictors of absconding behavior*

**Step**	**Variable**	**B**	**Wald**	***p***	**OR (95% C.I.)**
0	Intercept				
1	Diagnosis				
	Comorbid psychosis and substance use	.87	6.11	.01	2.38 (1.20-4.74)
	Other	.66	1.47	.27	1.93 (.67-5.62)
	History of absconding attempts	.41	1.55	.21	1.51 (.79-2.87)

*Note.* For diagnosis, primary psychosis (no comorbid substance use) is used as the reference group with indicator contrast coding.

*Model results: *χ*^2^ (3, *n* = 103) = 6.69, *p* = .082.

**Table 5:** Cox regression: risk-related predictors of absconding behavior*

**Step**	**Variable**	**B**	**Wald**	***p***	**OR (95% C.I.)**
0	Intercept				
1	Personality disorder indicated	.65	3.32	.07	1.92 (.95-3.88)
	PCL-R total score	-.01	.09	.76	.99 (.95-1.04)
2	HCR-20 total score	.11	9.13	.003	1.11 (1.04-1.20)

*Note*. PCL-R = Psychopathy Checklist, Revised; HCR-20 = Historical, Clinical, Risk Management-20.

*Model results: *χ*^2^ (3, *n* = 78) = 10.03, *p* = .018.

### Motivations for absconding

The comprehensiveness of the information collected regarding each absconsion in our sample allowed for a qualitative thematic analysis of motivation. Four distinct profiles of absconding behavior were identified: goal-directed, frustration/boredom, symptomatic/disorganized, and accidental.

#### Goal-directed

The 16 individuals who fell into this category were responsible for 19 events. They were likely to have voiced their desire to complete a specific directed activity, especially within the week prior to absconding, but at the time did not have the approval to do so. For example, after not being able to go for a haircut, one client promptly absconded and spent two hours in search of a barber. These clients showed no overtly concerning behavior during the month prior to absconding but did have several instances of minor non-compliance with privilege regulations (e.g., returning late). These absences were brief with the patient, upon accomplishing their goal, returning without difficulty. Seventeen (90%) of these incidents occurred during an unaccompanied pass, with only two occurring directly off a secure unit. This group also contained a subgroup of three individuals (four events) whose goal involved the specific desire to obtain substances, in one instance, *‘to celebrate my 60*^*th*^*birthday’*. Prior to absconding, these individuals were likely to engage in behavior indicative of planning (e.g., withdrawing money, selling belongings) but, unlike those in the broader goal-directed category, would not disclose intentions to staff. They were also identified as experiencing increased irritability and had staff-detected substance use in the month prior. Each of these four absconsions occurred during an unaccompanied pass with the client returning on their own, usually intoxicated.

#### Frustration/boredom

Frustration and/or boredom with the hospital, co-patients, staff and/or the forensic system was the most common absconding profile identified (52 incidents committed by 35 patients). During the month preceding the absconsion, patients frequently had privilege level changes, requests for privilege increases (without clear reasons), and generally difficult behavior suggestive of increasing frustration (e.g., involvement in verbal/physical aggression, medication non-compliance, substance use). Absconding ideation and discontent were seen in comments such as *‘needing a break’*, wanting to go home or *‘leave this place’*. Explicit statements noted by nurses, though less common, were very clear: *‘I am not coming back when I go out tomorrow’*, *‘going to elope once and for all’*, and *‘I will go AWOL when I get privileges back’*. Frustration regarding upcoming or unfavorable review board hearings/decisions was seen as an aggravating factor in a quarter of the absconding events within this group. Absconding was often described as impulsive in nature and accompanied by a high frequency of substance use. The majority of these incidents (69%) occurred while the patient was on an unaccompanied pass, either on hospital grounds or in the community. While absent these patients would sometimes contact the hospital to reiterate their frustrations about being *‘confined’,* or declare *‘I’m not coming back’*.

Nine of these cases, perpetrated by five different people, were further categorized as resulting from a general disregard for hospital rules. One individual, knowing he was already late, decided not to return to hospital for several days. Although these absconsions may appear unplanned, a recent pattern of non-compliance with privileges (e.g., returning late, overuse) combined with a history of absconding suggested otherwise. These individuals returned at their leisure, often minimizing the situation (*‘I wasn’t gone for long’*), externalizing blame (*‘I’m my brother’s responsibility’*), or citing a misunderstanding as a defense for their behavior.

#### Symptomatic/disorganized

In 28 absconding events the 20 patients responsible appeared to act in response to auditory hallucinations or delusional beliefs. In the month prior, these patients were experiencing active symptoms of their illness with notable mental status instability, medication changes, and/or missed medication. Reporting absconding ideation on multiple occasions to staff was common (e.g., *‘the voices are telling me to run away’*). This group had the highest proportion of clients absconding while under direct staff supervision. Specifically, 12 of these events (43%) occurred either through absconding directly off of the unit or running away during an accompanied pass. For example, one patient exclaimed *‘I get go’* before running, in order to avoid ‘*harmful vapors on the unit’*.

A subset of the absconding events from this group (*n* = 13; 7 individuals), were seen as stemming from goal-directed behavior directly linked to active psychosis. These incidents were preceded by multiple medication changes and missed dosages. Clients in this group often had several absconding incidents within a short time period (3-5 months), all with the objective of completing the same stated activity. One individual continuously stated he *‘had to go to work’*, upon returning from three separate absconsions and despite not having a job. A female patient, responsible for five separate incidents, was hoping to abscond *‘once and for all’* to be with the man she *‘loves’*.

#### Accidental/no intent

Three absconding incidents committed by three patients were identified as accidental. These patients lost track of time or encountered situations beyond their control, had no recent history of absconding and did not experience any concerning events in the month prior to the incident. These cases were of short duration, rare, and were referred to by nursing staff as *‘unintentional’*.

Lastly, we compared each of the motivational groups across the demographic and clinical variables listed in Table 3 (and excluding the Accidental group due to the small number of patients within this category). These findings should be viewed as preliminary and in need of replication due to the small numbers of patients falling into each motivational category. As shown in Table 6, patients who absconded in a goal-directed manner had spent comparably more time under the ORB (*F* [2, 51] = 2.59, *p* = .085), and also trended towards having higher PCL-R scores (*F*[2,35] = 2.65, *p* = .085). Furthermore, as would be expected, there was a higher proportion of patients with a primary psychotic disorder in the Symptomatic/Disorganized motivational group, while those with a comorbid substance use disorder were overrepresented in the Frustration/Boredom group (*χ*^2^ [1, N = 48] = 5.97, *p* = .051). This group was also judged to have significantly higher risk scores on the HCR-20 (*F* [2, 50] = 3.28, *p* = .046).

**Table 6:** Characteristics of absconders based on primary motivation

	**Goal-directed**		**Frustration/boredom**		**Symptomatic/disorganized**	
	**(*N*** **= 13)**		**(*N*** **= 25)**		**(*N*** **= 16)**	
	**M**	**SD**	**M**	**SD**	**M**	**SD**
Age	42.46	12.47	39.00	10.09	38.44	2.75
Days under ORB^†^	2911.00	2142.30	1811.16	1391.54	1801.63	1053.90
PCL-R total score^†^	21.07	4.71	17.81	8.01	14.71	3.85
HCR-20 total score^†^	25.62	3.40	27.42	4.58	23.31	6.41
	N	%	N	%	N	%
Sex (male)	11	84.6	19	76.0	12	75.0
Ethnicity						
Caucasian	9	69.2	12	48.0	6	37.5
Afro-Caribbean	3	23.1	9	36.0	5	31.3
Asian	0	0.0	2	8.0	5	31.3
History of absconding (yes)	9	69.2	12	48.0	10	66.7
Prior absconding attempts (yes)	4	30.8	7	28.0	5	31.3
Diagnosis^†^						
Primary psychotic	3	23.1	5	20.0	9	56.3
Psychosis + substance abuse	7	53.9	18	72.0	6	37.5
Personality disorder indicated (yes)	6	46.2	12	48.0	5	31.3
Index offense						
Violent	7	53.9	18	72.0	13	81.3
Non-violent	3	23.1	4	16.0	2	12.5
Sexual	3	23.1	3	12.0	1	6.3

^†^For Days under ORB, *F* (2, 51) = 2.59, *p* = .085; for PCL-R total score, *F* (2, 36) = 2.65, *p* = .085; for HCR-20 total score, *F* (2, 50) = 3.28, *p* = .046; for Diagnosis, χ^2^ (1, *N* = 48) = 5.97, *p* = .051.

## Discussion

Absconding from forensic psychiatric units is an issue which causes significant concern, not only in the community, but on hospital units. Despite findings in the literature, including results from the current study, to suggest that these incidents are relatively low in frequency and risk, they are perceived as significant breaches of public confidence and hospital oversight. It is therefore imperative to examine absconding events in greater depth to provide empirical evidence as to the characteristics of individuals who abscond from secure settings, as well as the predictors of and circumstances surrounding the incidents. This will facilitate accurate assessments of risk prior to granting a person leave, as well as the implementation of effective risk management interventions that address the specific circumstances and motivations of absconding events.

We employed an inclusive definition of absconding to capture all relevant dimensions of this behavior. The rate of absconding we detected (14.4%) was somewhat higher compared to prior studies, particularly those coming from secure settings, but here it is relevant to note that this study represents one of the first to examine this behavior comprehensively in a rehabilitating forensic sample, located in a hospital within a major urban center. We found that absconding events were generally of brief duration, and that the ultimate level of public endangerment posed by those who absconded was low. Specifically, no member of the public was harmed and no new criminal charges arose. The absence of adverse outcomes does not represent grounds for satisfaction, however, though it does suggest that persons being given privileges (such that a greater opportunity existed for them to abscond) did not present an imminent risk for violence to others or themselves. Beyond overt public safety risks such as when a patient reoffends in the community, there is a corrosive effect of absconding, a slowing of progress and delayed progression through rehabilitation goals, including reputational risk to the hospital.

Over half of all absconding patients returned on their own, with smaller proportions returning with the assistance of police (28%), family (7%), or hospital staff (6%). With respect to the duration of absconding events in this sample, it is comparable with what has been reported in the literature, but somewhat lengthier than the data from secure hospitals in the U.K. where many patients were reported to be apprehended within minutes of fleeing [10]. This difference is likely reflective of the fact that the majority of absconding events occurring in most samples, including this one, arose after the patient had already been granted leave from the unit or hospital. There will be a natural time delay in apprehending a person on an unescorted pass in comparison to patients who flee while under direct staff supervision. It may also relate to the relative ease geographically to gain access to the wider community in this (urban) setting.

The most striking difference between patients who had versus had not absconded was that the former group had spent significantly more time under the auspices of the forensic mental health system. Further, within the absconding group there were often significant time lapses between the date of admission for the index hospitalization and the date of first absconsion. This is consistent with at least one study conducted in a secure setting [11] where the average length of stay prior to a patient’s first absconsion was two months, and where the majority of events occurred within six or more months of admission. In contrast, the literature from non-forensic populations documents that absconding events tend to occur sooner, often within days or weeks of admission [23]. Unlike admissions to acute psychiatric units, entry into the forensic system is different in that many patients anticipate hospitalization that can last several years. As individuals come to understand and experience the often lengthy constraints on their liberty, even in the face of self-perceived improvements in their mental state, absconding events may become more frequent.

Consistent with the extant literature across civil and forensic samples, patients in this study who absconded were more likely to have a history of problematic substance use, as well as a history of attempted absconding (although this latter variable did not emerge as a unique predictor in the context of regression analysis). Indeed, the finding that one-third of all absconding events in this study involved the use of substances while absent from hospital, in violation of conditions specified in patients’ review board dispositions, suggests that this is a salient variable in the clinical picture of these patients.

Patients who absconded were also estimated to be at higher risk for future violence, as indicated on a structured professional risk assessment measure completed by the individual’s treatment team. Use of structured professional judgment tools such as the HCR-20, while designed to inform judgments of risk for future violence, may also be of use in assessing individuals who are at risk for absconding. This is clinically intuitive, given that absconding events often appear to reflect general noncompliance, impulsivity, active psychiatric symptoms, or an antisocial orientation, which are themselves risk variables identified in the HCR-20. Further, the HCR-20 has been shown to be useful in predicting and managing risk for a wide array of adverse outcomes (e.g., non-violent reoffending, hospital readmission; [35,36]), suggesting that it is not limited to interpersonal violence. That said, it is also the case that a tool such as the HCR-20 may be of limited utility in making predictions about violent behavior occurring during an absconding event. This is largely due to the fact that the base rate of violence during an absconsion tends to be extremely low, and was effectively nil in the current study. On the other hand, risk management strategies based on the HCR-20, designed to prevent and reduce the likelihood of harm, would conceivably be relevant across authorized and unauthorized community access scenarios.

We found that the majority of absconding events were characterized by expressed feelings of boredom and frustration. In their interviews with 52 patients residing on acute psychiatric admission wards in London, Bowers and colleagues [8] found boredom to play a salient role in patients’ decisions to abscond, alongside other variables such as feeling frightened and confined, or needing to complete tasks and household responsibilities. Interestingly, and in contrast to prior work in non-forensic samples [30], we did not find that fear or safety concerns played a significant role in patients’ decisions to abscond. It is likely that being in the forensic system confers particularly strong motivations related to feelings of frustration and despair when faced with a lengthy hospitalization and the curtailment of liberties in the community [11]. This may be further compounded by the fact that expressed feelings of frustration and boredom are challenging clinical issues for treatment teams to address. Nevertheless, the current findings suggest that larger systemic and environmental issues could be examined so as to create a consistent, transparent and respectful milieu which can contribute to a sense of legitimacy the individual attributes to their detention.

The next most common motivating influence we found in this sample pertained to absconders’ psychiatric symptoms. Individuals in this group appeared to act in response to auditory hallucinations or delusional beliefs, sometimes pursuing ostensibly goal-directed behavior directly linked to active psychotic symptoms. This aligns partially with the motivations reported by some of the patients in the Bowers et al. [8] study, but differs in that patients reporting psychotic motivations in that study concurrently expressed non illness-related reasons for absconding. Our finding also contrasts with the study by Dolan and Snowden [11] in which only one individual expressed a psychotic motivation for their behavior. In the current study, there appeared to be clear proximal risk indicators for this group: in the month prior these patients were experiencing active symptoms of their illness with notable mental status instability, medication changes, and/or missed medication. These variables indicate important changes in patient’s level of clinical stability, and may also reflect deteriorations in the therapeutic relationship between the patient and the treatment team. These variables thus appear relevant to the assessment of risk for absconding, particularly among this subgroup of patients, and could well serve as choice points for intervention prior to an individual being granted privileges to leave the unit.

In the “goal-directed” profile, individuals were likely to have voiced their desires to complete a specific directed activity, but did not have the approval to do so at the time. During the absconsion, most of these patients completed their self-identified goal and then returned to the unit without difficulty. This group appears similar to Bowers et al.’s [8] patients who absconded due to needing to complete everyday household tasks and chores. A review of the documentation surrounding these events did not always provide the reasons for the refusal of passes so that the individual could accomplish their goal. It is also notable that in approximately one-third of incidents, interspersed across the motivational groups, the patient openly expressed thoughts or intentions to abscond from hospital. These statements require further examination as proximal risk indicators of absconding. Here, conducting interviews with clinical and front-line staff may be particularly informative in determining how or why breakdowns in communication occur between staff and patients, and interventions that can be implemented to improve this.

At present, there exists no structured decision-making tool to assess a patient’s risk for absconding. Hilterman, Philipse, and Graaf [37] published a tool assessing the risk of violence on discharge or unauthorized leave in the community, but not of the risk of absconding itself. This is problematic, particularly in light of the finding that forensic hospitals show considerable variation with respect to the criteria they rely on to make decisions about a patient’s readiness for community access [38]. Bowers, Alexander, and Gaskell [39] suggested identifying patients at higher risk for absconding based on the presence of specific variables shown to be predictive of absconding in the research base. Then, intensified resources such as one-to-one nursing time and increased family visits are encouraged to promote the open discussion of worries or concerns that might be further contributing to the person’s risk of absconding. One of the major benefits of this study is the identification of four broad motivations for absconding, which can further facilitate efforts to target those variables that are associated with absconding risk (e.g., mental status and medication effectiveness in the symptomatic group) in clinical decision-making and care planning.

There are limitations to the current study. First, our measure of absconding rate was a coarse one; although it is consistent with prior studies and recommended as one of the better options available [1,34], we were unable to supplement it with more fine-grained measures (e.g., number of absconsions on unsupervised day passes divided by the total number of day passes granted) due to the unavailability of this type of data. Second, information which formed the basis of our analyses were taken from the electronic health record, and supported by other legal documentation. We did not specifically interview patients about why they absconded. Therefore, the available information was that which staff documented; interviews with absconders and front line staff may have assisted in providing more detailed information surrounding the motivational aspect of the behavior. Further, this was a retrospective study, and we did not examine the impact of absconding prospectively (e.g., in terms of length of stay in the system, clinical progress, or experiencing other adverse outcomes such as violence or victimization in the community). Additional longitudinal analyses may be able to detect other trends in absconding behavior and identify the impact of changing political or environmental issues on this behavior over time. Importantly, our sample size was modest, and became smaller when conducting analyses based on motivation. Consequently, power to detect effects, particularly those small in size, was also modest, and underscores the need to replicate the current findings in larger samples of forensic patients.

Given that so few studies have examined absconding events from forensic settings, further research is needed to replicate the significance and clinical utility of the variables identified in this study that were associated with a heightened likelihood of absconding. Additionally, replication of the motivational subtypes using interview-based techniques is needed. Further examination of interventions which address the specific variables known to influence absconding behavior is also essential; as noted by Bowers and colleagues [1], “there are no thoroughly convincing, well designed, rigorously carried-out trials of interventions to reduce absconding” (p. 350). The current study can provide the basis for an intervention study tailored to the clinical profiles and reported motivations of those who abscond from forensic rehabilitation settings. In fact, as a consequence of this work, we made substantial changes to our program’s policies outlining the granting of privileges and we will report the outcome of this policy implementation in a separate paper.

## Conclusion

In contrast to public perception, the absconding events identified in this study were found to be generally brief and did not involve violence. Despite this, it is important to recognize that many of these patients had committed an index offense involving serious violence, and were also judged to be at elevated risk for future violence. Therefore, while no member of the public was harmed, the risk to public safety was indeed present, and affirms the clinical relevance of absconding behaviors and the importance of further research into their characteristics and determinants. Patients who absconded were significantly more likely to have a co-occurring substance use disorder, as well as be estimated as posing a higher risk for violence. They had spent significantly more time under the auspices of the forensic mental health system, but were less likely to have committed an index offense comprising serious violence. Our analysis of patient-reported motivations revealed a wide-spread sense of boredom and frustration with the forensic system, but also pointed to some heterogeneity in the primary motives underlying the decisions to abscond. The combination of empirically derived risk factors that are found to heighten the likelihood of absconding with patient-reported motivations can be used to further inform and refine decision-making around security needs and granting leave for forensic patients, with the ultimate goal of reducing the incidence of absconding.

## Endnotes

^a^We had exhausted our list of appropriate female matches and so there is one female in the absconding group with no matched control.

^b^For both the absconding and control groups, time under the ORB was calculated as the difference between the person’s original disposition date (i.e., the initial hearing date that brought them under the Review Board) and the end date of the current study (December 31, 2011).

^c^Beginning in approximately 2010-2011, these risk assessments were being completed on all patients in the program on an annual basis. However, many patients included in the current sample (*n* = 36) did not have HCR-20 scores prior to their first absconsion; therefore, we utilized the most proximal post-absconsion score (for which everyone had) in order to ensure consistency across the sample. Three-quarters of the sample had an HCR-20 score generated within 12 months of their first AWOL (range = 0-23 months). For the control group, the HCR-20 scores were taken from the same time window (i.e., 2011-2012).

## Competing interests

The authors declare that they have no competing interests.

## Authors’ contributions

TW and SP conceptualized the aims and design of the study. SF carried out the data collection, while TW, SP and SF contributed to the data coding. SP performed the statistical analyses. All authors contributed to the interpretation of the data and the study write-up. TW, SP, and SF drafted the manuscript, and AIFS revised it critically for intellectual content. All authors read and approved the final manuscript.

## Pre-publication history

The pre-publication history for this paper can be accessed here:

http://www.biomedcentral.com/1471-244X/14/91/prepub
